# The Non-Essential Mycolic Acid Biosynthesis Genes *hadA* and *hadC* Contribute to the Physiology and Fitness of *Mycobacterium smegmatis*

**DOI:** 10.1371/journal.pone.0145883

**Published:** 2015-12-23

**Authors:** Stevie Jamet, Nawel Slama, Joana Domingues, Françoise Laval, Pauline Texier, Nathalie Eynard, Annaik Quémard, Antonio Peixoto, Anne Lemassu, Mamadou Daffé, Kaymeuang Cam

**Affiliations:** 1 Centre National de la Recherche Scientifique, IPBS, UMR 5089, F-31077 Toulouse, France; 2 Univ. Toulouse, UPS, F-31000 Toulouse, France; University of Padova, Medical School, ITALY

## Abstract

Gram positive mycobacteria with a high GC content, such as the etiological agent of tuberculosis *Mycobacterium tuberculosis*, possess an outer membrane mainly composed of mycolic acids (MAs), the so-called mycomembrane, which is essential for the cell. About thirty genes are involved in the biosynthesis of MAs, which include the *hadA*, *hadB* and *hadC* genes that encode the dehydratases Fatty Acid Synthase type II (FAS-II) known to function as the heterodimers HadA-HadB and HadB-HadC. The present study shows that *M*. *smegmatis* cells remain viable in the absence of either HadA and HadC or both. Inactivation of HadC has a dramatic effect on the physiology and fitness of the mutant strains whereas that of HadA exacerbates the phenotype of a *hadC* deletion. The *hadC* mutants exhibit a novel MA profile, display a distinct colony morphology, are less aggregated, are impaired for sliding motility and biofilm development and are more resistant to detergent. Conversely, the *hadC* mutants are significantly more susceptible to low- and high-temperature and to selective toxic compounds, including several current anti-tubercular drugs.

## Introduction

*Mycobacterium tuberculosis*, the etiological agent of tuberculosis, infects one-third of the world population with 9 million new cases and 1.5 million people dying each year from this disease [1]. This failure is primarily due to the capability of the pathogen to develop a non-replicating persistent drug-tolerant form [2,3,4] and to the outbreak of multi-drug and total-drug resistant strains [5,6,7]. The main reason to the development of resistance is the poor compliance with medical treatments due to the side-effects on everyday wellbeing and the duration of treatments [8]. There is therefore an urgent need finding new drugs with highly short-time efficiency [9] or increasing the efficiency of existing drugs [10,11].

*M*. *tuberculosis* has a unique cell envelope, with a highly efficient permeability outer membrane barrier crucial to its viability and virulence. This so-called mycomembrane is composed of long-chain (up to C100) fatty acids, called mycolic acids (MAs), whose biosynthesis is targeted by several major anti-tubercular drugs [12,13]. In mycobacteria, the synthesis of MAs involved two distinct Fatty Acid Synthases (FAS), *i*.*e*. the eukaryote-like multifunctional single protein FAS-I and the bacterial-like multi protein complex FAS-II [13]. The HadA-HadB and HadB-HadC heterodimers of the FAS-II complex perform a key dehydration reaction [14,15]. Enzymatic assays have shown that HadA and HadC subunits are involved in the substrate specificity, bringing either short-/medium- or long-size substrates to the catalytic activity of HadB, respectively [14]. The *hadABC* knock-out mutant in *M*. *tuberculosis* was shown to be non-viable [14] and comprehensive transposon mutagenesis has concluded that *hadA* and *hadB*, but not *hadC*, are essential for cell viability [16,17], although so far only the essentiality of *hadB* in *M*. *smegmatis* has been confirmed [15], while the non-essentiality of *hadC* has been shown in *M*. *tuberculosis* [18]. Nevertheless, the key dehydration step for the synthesis of MAs has stimulated the search for drugs that would target the Had enzymes. Indeed, two anti-tubercular drugs used in the sixties, Thioacetazone and Isoxyl, have recently been shown to target HadC and HadA [19]. Although both drugs are barely used because of either a low efficacy (for Isoxyl [20] or toxic side-effects (for Thioacetazone [21]) they underscore the fact that the proteins Had are druggable targets for fighting tuberculosis.

*M*. *smegmatis*, a fast-growing and safer-to-handle species, has been widely used as a surrogate of the highly pathogenic *M*. *tuberculosis*. This is based on the assumption of the conservation of the basic functions in both species, such as the building of the mycomembrane. As a consequence, a better understanding of *M*. *smegmatis* physiology would benefit to the knowledge of *M*. *tuberculosis* physiology. Notwithstanding *M*. *smegmatis* belongs to the nontuberculous mycobacteria (NTM) complex and as such *per se* is also an opportunistic pathogen for humans and animals [22,23,24,25]. Therefore any further understanding of *M*. *smegmatis* might also give new hints to better fight against hardly-cured diseases due to NTM. In this study, we decipher the respective biological role of the HadABC dehydratase subunits and show that *M*. *smegmatis hadA* and *hadC* genes are not essential for cell viability but play a major role in the physiology and adaptive response of the bacteria.

## Materials and Methods

### Bacterial strains, plasmids and growth conditions

Strains and Plasmids used in this study are listed in Table 1. For liquid cultures, mycobacteria strains were grown in Middlebrook 7H9 medium (Difco) containing 0.05% Tween-80, 0.2% glycerol, 10% ADC (Difco) and the appropriate antibiotics (Kanamycin 37.5 μg/ml, Hygromycin 150 μg/ml). For solid medium, Tween-less Middlebrook 7H10 broth supplemented by 0.5% glycerol and 10% OADC (Difco) was used. When required, Zeocin was added at 15 μg/ml. For *E*. *coli* growth, Luria-Bertani medium (Invitrogen) was used with antibiotics when required (Kanamycin 37.5 μg/ml, Hygromycin 150 μg/ml). To induce the *tetROp* promoter from the pGBT plasmid and its derivatives, Tetracycline (20 ng/ml) was also added to the liquid and solid media.

**Table 1:** List of strains and plasmids.

Bacterial strains	description	sources
mc2 155	*M*. *smegmatis mc* ^*2*^ strain	[26]
Δ*hadC*	native copy of MSMEG_1342 replaced by *Sh ble* (ZeoR)	This work
Δ*hadABC* pABC	native copy of MSMEG_1340/1341/1342 operon replaced by *Sh ble* (ZeoR), and carrying the pABC plasmid	This work
MC1061 *recA*	*E*. *coli* cloning strain	Our collection
**Plasmids**		
pJV53	Kan^R^, Plasmid expressing the recombineering proteins	[27]
pJV53::hyg	Hyg^R^, Hygromycin resistant derivative of Plasmid pJV53	V. Malaga
pGBT	Kan^R^, *C*. *glutamicum tetRO* promoter cloned in pGB9.2	[28]
pMVZ261	Zeo^R^, source of the *Sh ble* gene	G. Etienne
pC	Kan^R^, native copy of MSMEG_1342 cloned in pGBT	This work
pABC	Kan^R^, native copy of MSMEG_1340/1341/1342 in pGBT	This work
pAB	Kan^R^, native copy of MSMEG_1340/1341 cloned in pGBT	This work
pBC	Kan^R^, native copy of MSMEG_1341/1342 cloned in pGBT	This work
pB	Kan^R^, native copy of MSMEG_1341 cloned in pGBT	This work

### DNA manipulation

Molecular biology materials were used as recommended by the manufacturers: DNA purification (Quiagen), enzyme restrictions and T4 DNA ligase (Fermentas and Biolabs), PCR with the phusion polymerase (Finnzyme), and pJET1.2 cloning kit (Fermentas). DNA inserts were checked by sequencing (MillGen).

### Construction of deletion mutants

KO-mutants were generated with the recombineering system [27,28], with slight modifications [28]. To delete the whole *hadABC* cluster, co-transformation was done with 100 ng of plasmid DNA along with 100 ng of AES (allelic exchange sequence), and selection made on Zeocin, Kanamycin and Tetracycline containing medium. PCR on lysates of recovered clones were performed to check for the replacement of the target sequence by the Zeocin resistant cassette.

### Drugs and temperature susceptibility assays

Cultures at OD_590_ ~4–5 of the different strains were adjusted to the same OD then serially diluted. 5 μl of each dilution (starting OD_590_ 0.2) was spotted on 7H10-based medium containing OADC, glycerol, Tetracycline (20 ng/ml) and Kanamycin (37.5 μg/ml). When required, drugs were added to the medium: Rifampicin (2 μg/ml), Isoniazid (5 μg/ml), Ethionamid (10 μg/ml), Ethambutol (5 μg/ml) and Vancomycin (1 μg/ml). After 4–5 days at 37°C (for drugs testing) or at 30°C, 37°C and 42°C (for temperature testing), CFUs were counted.

### Susceptibility to SDS

Cultures were grown to OD_590_ ~ 0.6–0.8 in 7H9 medium + ADC + glycerol + tween + Kanamycin + Tetracycline, harvested, washed once with 7H9 + tween, and suspended in an equal volume of 7H9 + glycerol + Kanamycin + Tetracycline + tween. Then, each preparation was adjusted to OD_590_ 0.2 and SDS added to 0.1% final. After 65 min, aliquots were serial diluted and spotted on growth plates. Survival rate was estimated by counting the CFUs after incubation for 3–4 days at 37°C.

### Sedimentation assays

Cultures at OD_590_ ~4–5 of the different strains in 7H9 + ADC + glycerol + tween + Kanamycin + Tetracycline, were adjusted in triplicate to OD_590_ ~ 1 and kept unshaken at 37°C. At 3 and 22 hours, the upper 1 ml was removed for OD measurements.

### Biofilm formation

Exponential phase cultures (OD_590_ ~1) in 7H9 medium + ADC + glycerol + tween + Kanamycin + Tetracycline, were diluted (1:100) in standard Sauton’s media [29] containing Kanamycin and Tetracycline. Then 4.5 ml samples were dispensed into 12 wells plates (3 wells/strain). After being wrapped three times with parafilm, the plates were incubated at 37°C for one week.

### Nile Red accumulation

The experiment was performed as previously described with slight modification [30]. Strains were grown as a cell layer on solid 7H10 medium containing glycerol, ADC, Kanamycin and Tetracycline. Cells were scrapped and resuspended in PBS buffer (containing 25 mM glucose). Each suspension was adjusted to OD_590_ 0.5, then Nile Red added (4 μM final). Fluorescence at 610 nm (excitation at 533 nm) was measured in technical triplicate in a 96 wells plate (incubated at 37°C) with the Clariostar reader (BMG).

### Total RNAs preparation

Cultures were grown to OD_590_ ~ 0.6–0.8 in 7H9 medium + ADC + glycerol + tween + Kanamycin + Tetracycline. Total RNA was extracted using the RNeasy kit (Qiagen) following manufacturer’s instructions with slight modifications. Briefly, 15 ml of cultures were centrifuged for 5 min at 1,600 g, the pellet suspended in 1.2 ml of 0.1% β-mercaptoethanol containing RLT lysis buffer along with 0.1 mm-diameter glass beads. Cells were lysed by two 120 sec pulses at full speed in a bead-beater device. The sample was centrifuged 30 sec at 14,800 g. One volume of absolute ethanol was added to the filtrate, and total RNA purified with an RNeasy column following the manufacturer’s procedure. RNA sample was treated twice for 45 min with successively 3U and 2U of Turbo DNase (Turbo DNA *free* kit-Ambion). Biological triplicates were performed for each condition.

### Analysis of Mycolic Acids. Cultures were grown to stationary phase in 7H9 medium + glycerol + Kanamycin + Tetracycline

Whole cells or bacterial residues obtained after lipid extraction with organic solvents [31] were saponified by a mixture of 40% KOH and methoxyethanol (1:7, v/v) at 110°C for 3 h in a screw-capped tube. After acidification, fatty acids were extracted with diethyl ether and methylated with an ethereal solution of diazomethane[32]. The mycolate patterns of the strains were determined by HPTLC (High Performance Thin-Layer Chromatography) on HPTLC Silica Gel 60 (Merck), using a mixture of petroleum ether/diethyl ether (9:1, v/v, five runs) as eluent. Revelation of lipid spots was performed by immersion of the plate in a solution of rhodamine. The various classes of mycolates, alpha-, alpha’, epoxy-mycolates (α, α', E, respectively) and the compound X were quantified by absorption measurement at the specific wavelength with TLC Scanner 3 using wincats software.

Matrix Assisted Laser Desorption Ionization-Time of Flight Mass Spectrometry (MALDI-TOF MS) was performed in reflectron mode, using the 5800 MALDI-TOF/TOF Analyzer (Applied Biosystems/ABsciex) equipped with a Nd:YAG laser (349 nm wavelength). A total of 2,500 shots were accumulated in positive ion mode. Lipid samples were dissolved in chloroform and were directly spotted onto the target plate as 0.5 μl droplets, followed by the addition of 0.5 μl of matrix solution (10 mg of 2,5-dihydroxybenzoic acid [Sigma-Aldrich]/ml in CHCl_3_/CH_3_OH, 1/1 [vol/vol]). Samples were allowed to crystallize at room temperature. Mass spectrometry data were acquired using the instrument default calibration [33].

For Nuclear Magnetic Resonance (NMR) analyses, compounds were dissolved in CDCl_3_/CD_3_OD (1/1, v/v, 99.8% purity, Euriso-top, CEA Saclay, France). 1D and 2D ^1^H- COSY 1H/1H (COrrelation SpectroscopY) experiments were conducted in the 600 MHz Bruker NMR spectrometer equipped with cryosonde. ^1^H chemical shifts are given in parts/million downfield from internal tetramethylsilane at 0 ppm. All experiments were recorded at 295° K without sample spinning. The Bruker pulse programs were used and optimized (pulse lengths and delays) for each one- or two-dimensional experiments. Data were analyzed using the TopSpin (Bruker BioSpin) software.

## Results

### The HadA and HadC subunits are dispensable to cell viability

**Fig 1:**
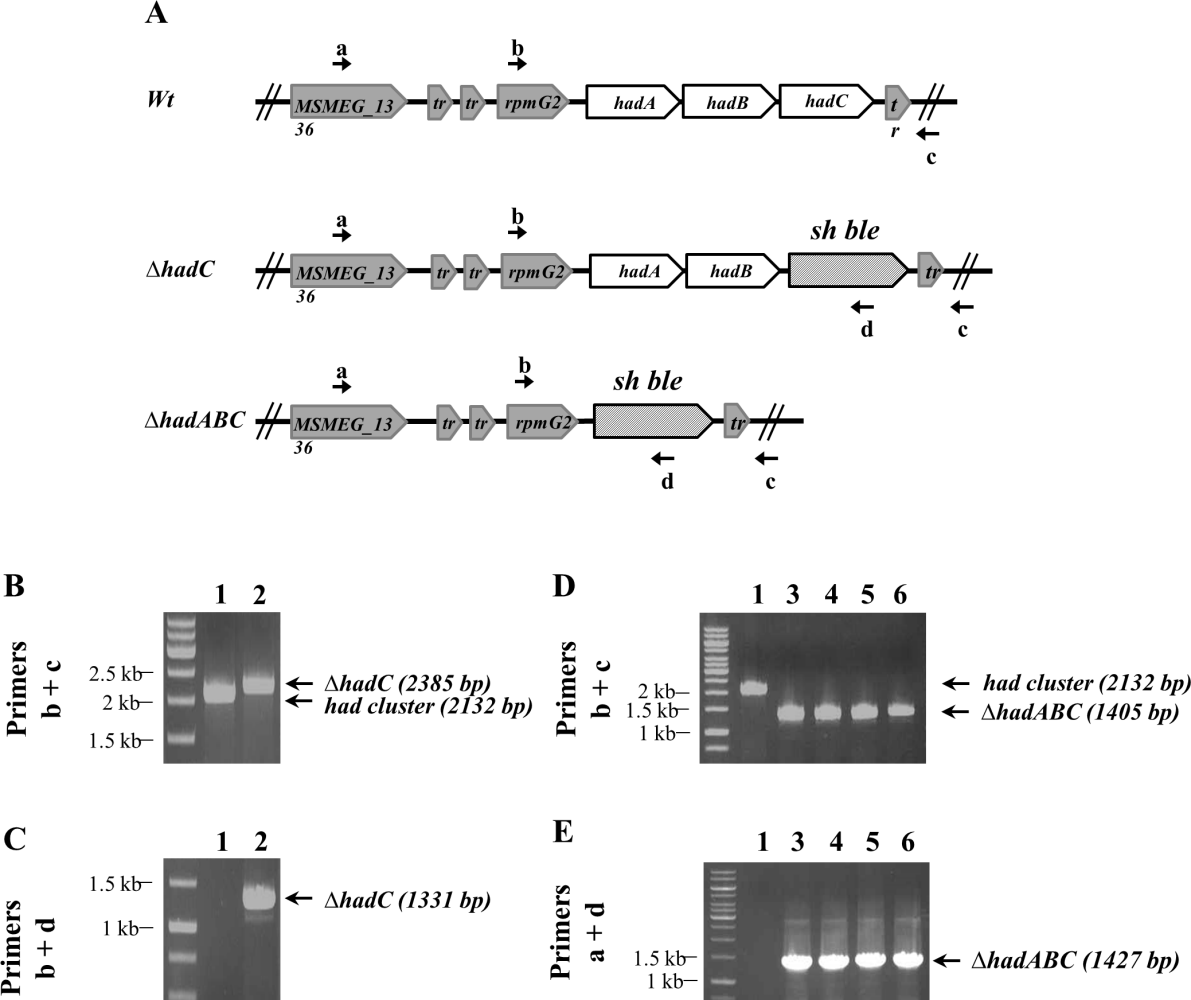
Construction of *M*. *smegmatis ΔhadC* and *ΔhadABC* strains by recombineering. (A) Genetic organization showing the replacement of the *hadC* gene and *hadABC* operon by a zeocin resistance cassette (*Sh ble*) and the primers a, b, c, d used for PCR verification of the constructions. (b+c) primer couple confirms the deletion of the *hadC* gene (B) and *hadABC* operon (D). Amplification with the (b+d) and (a+d) primer couples confirms the presence of the zeocin cassette in the *ΔhadC* (C) and *ΔhadABC* (E) strains, respectively. PCR was performed on cell lysates: *wt* (lane 1), *ΔhadC* (lane 2), *ΔhadABC* (lanes 3–6) carrying either the plasmid pABC (lane 3) or pAB (lane 4) or pBC (lane 5) or pB (lane 6).

In *M*. *tuberculosis*, the *hadA*, *hadB* and *hadC* genes may be co-transcribed from five promoters [28]. The synteny observed between *M*. *tuberculosis* and *M*. *smegmatis* in this region suggests that the operon organization is also conserved in *M*. *smegmatis* [28]. Comprehensive transposon mutagenesis in *M*. *tuberculosis* has concluded that *hadC* is not essential [16,17]. Accordingly we were able to delete *hadC* in *M*. *smegmatis*, by replacing the gene by a zeocin resistance cassette (Fig 1A–1C). To get further insights into the contribution of each of the *hadA*, *hadB* and *hadC* genes to the bacterial physiology, we attempted to generate mutants bearing various deletion combinations of the three genes. To avoid any polar effect of inactivating genes organized into an operon, this was done by co-transforming the *wt* strain with an allelic exchange sequence fragment to delete the whole *hadABC* operon and a plasmid expressing different combinations of the *had* genes. The set of plasmids included pGBT, the cloning vector, and pABC, pBC, pAB, pB expressing from a *tetROp* promoter either the whole operon or *hadB and hadC or hadA and hadB* or only *hadB*, respectively. Each co-transformation was independently repeated at least twice and consistently gave reproducible results. As expected, no co-transformant was obtained with the vector alone (pGBT), *hadB* being essential [15], whereas complementing with the pABC plasmid gave 10–15 colonies. With the same efficiency, plasmid pAB (*ΔhadC*) also gave co-transformants, in agreement with our ability to delete the chromosomal copy of the *hadC* gene. Interestingly, transformants were also obtained with plasmid pBC (*ΔhadA*), indicating that *hadA* was not essential either in *M*. *smegmatis*. More surprisingly, a comparable co-transformation efficiency was observed with plasmid pB (*ΔhadAC*), indicating that cells were viable despite the inactivation of both *hadA* and *hadC* (Fig 1). Therefore HadB was the only essential subunit of the HadA-HadB and HadB-HadC dehydratase complexes.

### Impact of HadC on the adaptive response to cold shock

**Fig 2:**
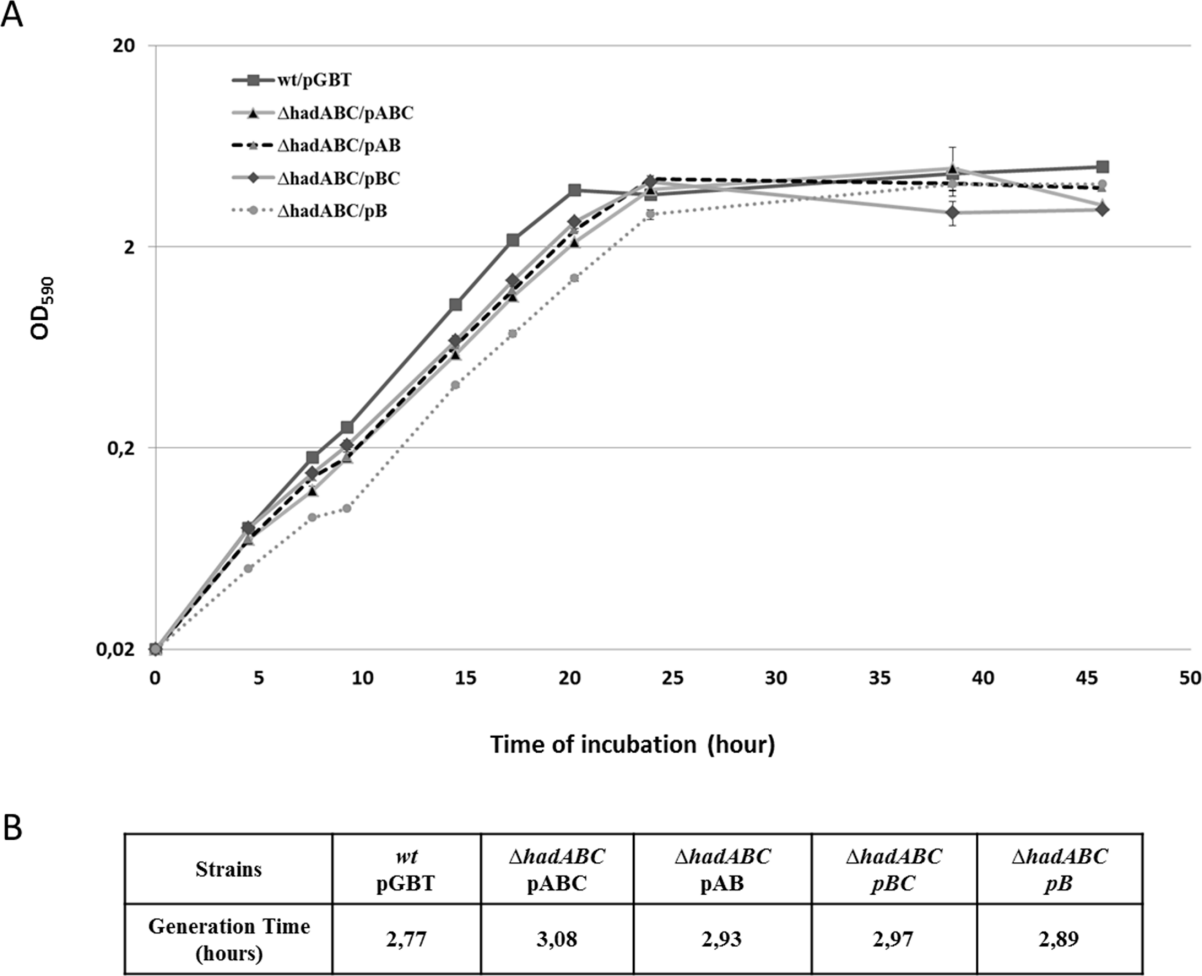
The inactivation of either *hadA*, *or hadC* or *hadAC* does not affect the growth of *M*. *smegmatis*. (A) Growth curves at 37°C in 7H9-based medium. Error bars represent standard deviations from three biological replicates. (B) Calculated generation time for each strain during exponential growth.

**Fig 3:**
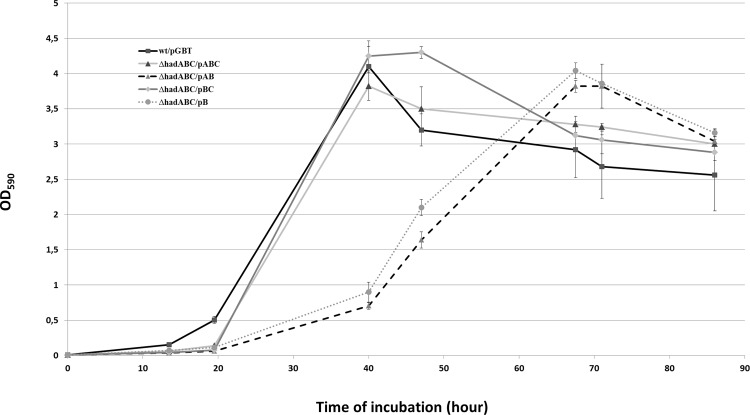
*hadC* is required for the adaptive response to cold-shock. Exponential phase cultures at 37°C were diluted 100-fold in 7H9-based medium kept at room temperature (and not in a 37°C pre-warmed medium as in Fig 2) then re-incubated at 37°C (time 0). Error bars represent standard deviations from three biological replicates.

As a first step to evaluate the contribution of each of the *had* genes to the fitness of the bacterium, the growth of the mutants was followed in 7H9-based liquid cultures. As shown in Fig 2A and 2B, the growth curves of the single (*ΔhadC and ΔhadA*, *i*.*e*. *ΔhadABC*/pAB and *ΔhadABC*/pBC, respectively) and double mutants (*ΔhadAC i*.*e ΔhadABC*/pB) were comparable to that of the *wt* reference strains (*wt*/pGBT and *ΔhadABC*/pABC), each strain reaching a similar plateau value with similar growth rates (Fig 2B). In contrast, when a cold-shock was applied, although a lag was observed for all strains before the growth resumed, the lag displayed by the *ΔhadC* (*ΔhadABC*/pAB) and *ΔhadAC* (*ΔhadABC*/pB) mutants lasted twenty hours longer (Fig 3). As the growth profile of the *ΔhadA* (*ΔhadABC*/pBC) was similar to that of the control *wt* strains, it was concluded that the longer lag phenotype of the *ΔhadC* and *ΔhadAC* strains was likely due to the inactivation of the *hadC* gene. Expectedly, the simple *hadC* deletion mutant (*ΔhadC*/pGBT) displayed a growth profile similar to that of *ΔhadABC*/pAB, a phenotype fully complemented by a plasmid expressing *hadC* (*ΔhadC*/pC) (data not shown). Therefore a functional HadC, although not essential, is required for the adaptive response of the bacterium to a cold-shock.

### Colony morphology and sedimentation velocity in the *had*A/*hadC* mutants

**Fig 4:**
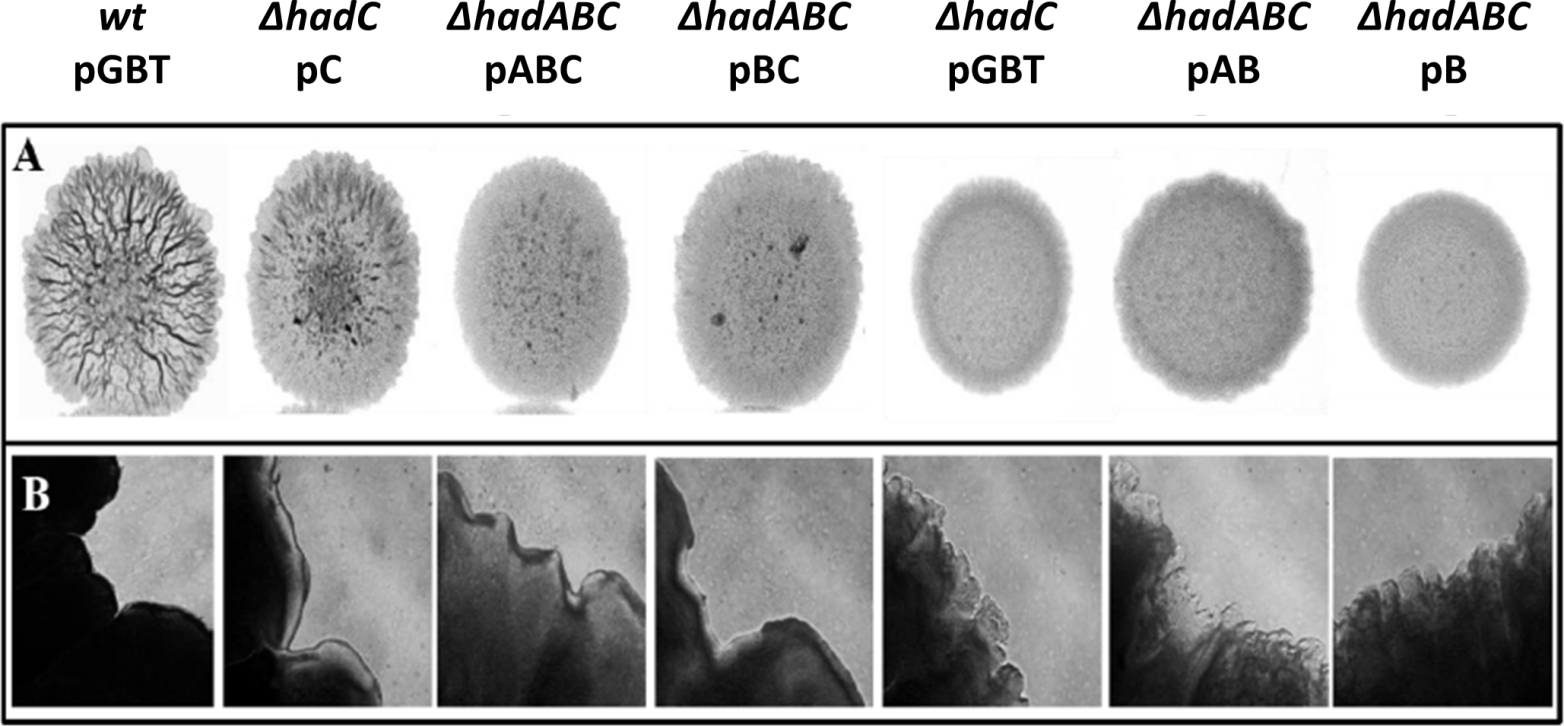
The colony morphology of the *had* mutants is altered. (A) Colony morphology of the various strains grown on 7H10-based medium. (B) Corresponding borders of the colony (10X-magnification), representative of five pictures taken at different locations.

**Fig 5:**
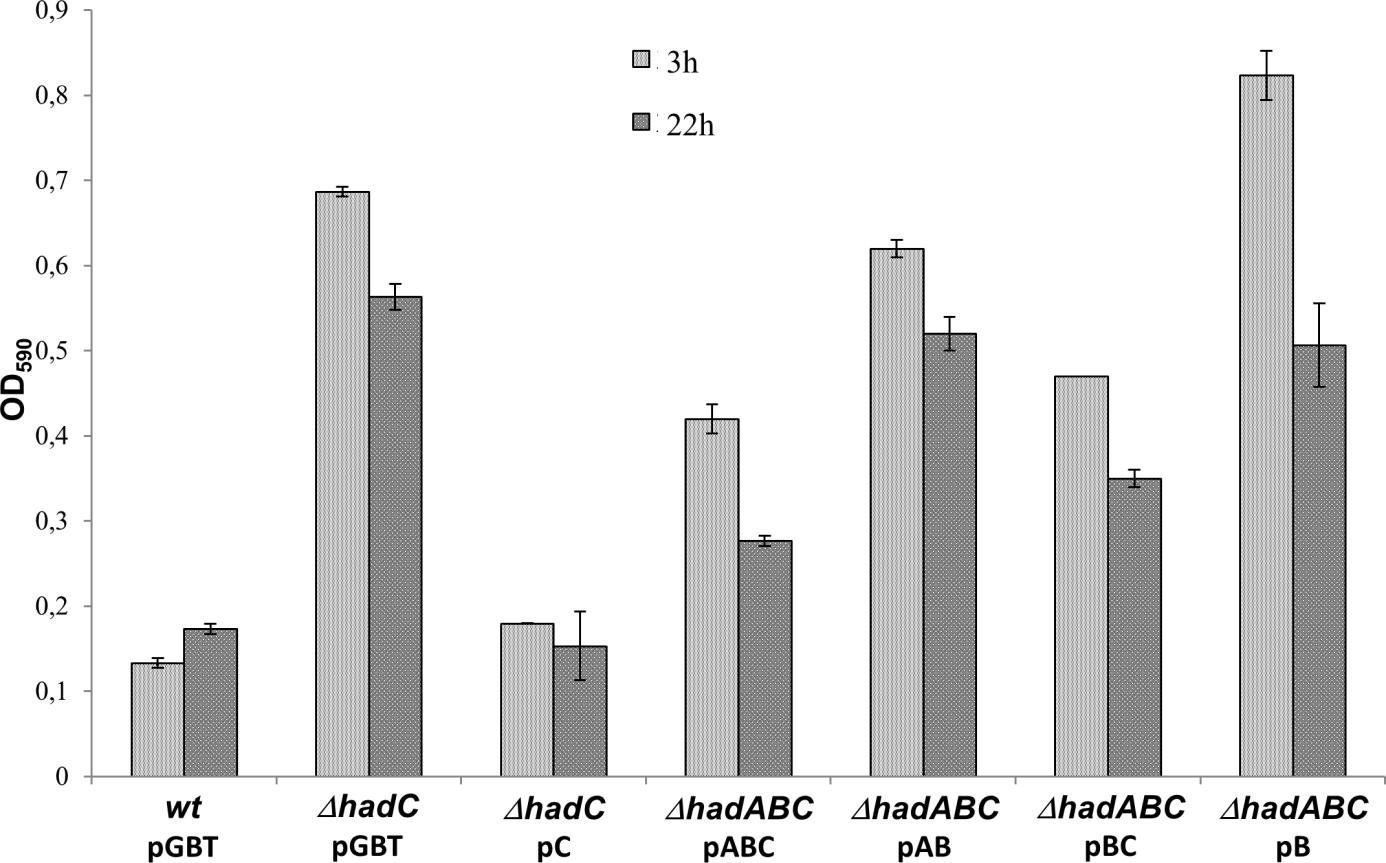
The inactivation of *hadC* reduces the cell sedimentation rate. Cultures at OD_590_ ~ 4–5 were adjusted at OD_590_ ~ 1 (in triplicate) in a 7H9 liquid medium containing Tween (0.05%) and Tetracycline (20 ng/ml) to induce the *tetROp* promoter, then kept at 37°C without agitation. Sedimentation rates were estimated by measuring the OD of 1 ml sample taken from the upper most layers at 3 and 22 hours. Error bars represent standard deviations.

The colony morphology of some of the mutants was readily distinguishable from that of the *wt*, as illustrated in Fig 4. The *wt* strain (*wt*/pGBT) formed rough colonies with ridges and well-delimited borders whereas the *hadC* mutant *(ΔhadC/pGBT*) formed smooth colonies with unstructured border. The introduction of a plasmid expressing the wild-type *hadC* allele from a TetR-regulated promoter (pC) partially restored the wild-type morphology *(ΔhadC/pC*). In the *ΔhadABC* background, the expression of the *hadABC* genes from the *tetRO* promoter (pABC) led to the formation of rough colonies with well delimited border, although the roughness was less pronounced than that of the *wt* strain (Fig 4). The absence of a full complementation in both *ΔhadC*/pC and *ΔhadABC*/pABC strains was likely due to a lower expression of the *had* genes from the *tetRO* promoter as shown by RT-qPCR (S1 Fig). The morphology of the *ΔhadABC*/pAB colonies (*ΔhadC*) as well as that of the double mutant *ΔhadABC*/pB (*ΔhadAC)* was smoother with more unstructured border than that of the appropriate reference strain (*ΔhadABC*/pABC). Considering that the morphology of the *ΔhadABC*/pBC colony (*ΔhadA*) was similar to that of the control *ΔhadABC*/pABC strain, we concluded that the alteration of the colony morphology was mainly due to the inactivation of *hadC*. Smooth colony morphology might be indicative of the alteration of the cell surface proprieties. Because cell sedimentation velocity is inversely correlated to the hydrophobicity of the envelope [34], measuring the rate of sedimentation could be a mean of underscoring the alteration of the envelope. When shacked cultures at OD_590_ 1 were kept unshaken for 3 h and 24 h, the ODs of the upper layer of the cultures were 0.7 and 0.6 respectively for the *ΔhadC* mutant, whereas those for the *wt* cultures dropped to OD_590_ ~0.15. The complementation of the mutant with the plasmid expressing *hadC* fully restored the wt phenotype (Fig 5). In the *ΔhadABC* background, similar results were obtained, *i*.*e*. the sedimentation velocity of the *ΔhadC* was slower (Fig 5, *ΔhadABC*/pAB). In agreement with the colony morphology phenotype, the sedimentation velocity of the *ΔhadA* mutant was similar to that of the *wt* strain (Fig 5, *ΔhadABC*/pBC), whereas that of the double *ΔhadAC* mutant (*ΔhadABC*/pB) was slower. Of note, the velocity of the double mutant was even slower than that of the single mutant *ΔhadC* at 3 h (*ΔhadABC*/pAB), indicating an additive effect of the *hadA* mutation on the phenotype of the *ΔhadC* strain. Therefore, altogether these results indicated that the HadC protein plays a major role in keeping the wild-type characteristics of the envelope, notably its hydrophobicity.

### The development of biofilms and the sliding motility are negatively affected by the inactivation of HadC

**Fig 6:**
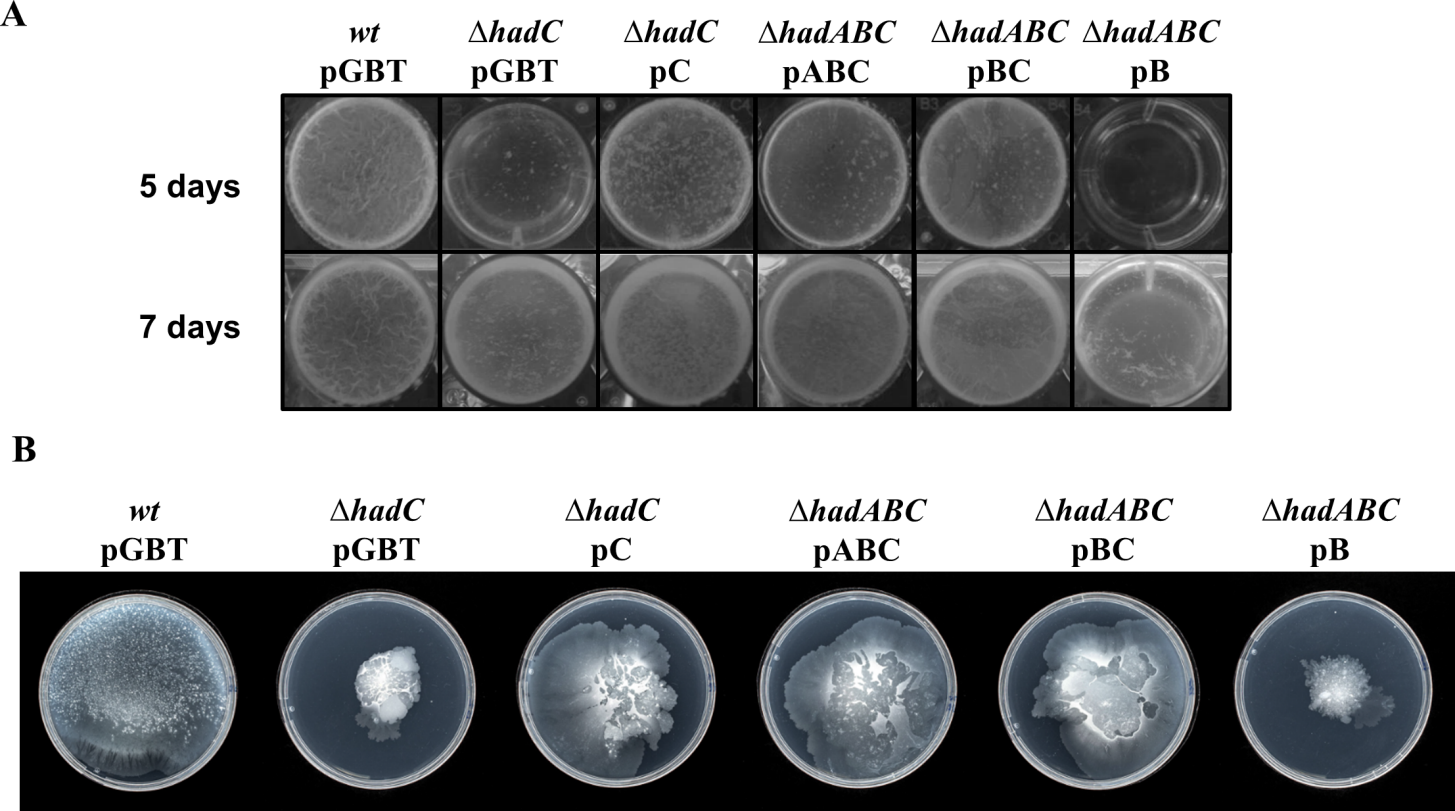
The inactivation of *hadC* compromises the development of biofilms and the sliding motility. (A) Biofilm formation was monitored after 5 and 7 days of growth. The picture is representative of 3 biological replicates. (B) The sliding motility was monitored after 7 days of incubation on a semi-solid 7H9 medium without any carbon source.

Lowering the hydrophobicity of the cell surface would negatively interfere with both the formation of biofilms and the sliding motility of *M*. *smegmatis* [35]. As shown on Fig 6A, the *hadC* mutation dramatically delayed the development of biofilms. At day 5 the *wt* (*wt*/pGBT, *ΔhadABC*/pABC) as well as the *ΔhadA* (*ΔhadABC*/pBC) strains displayed a visible pellicle, in contrast to any of the *ΔhadC* deletion bearing mutants (*ΔhadC/pGBT*, *ΔhadABC*/pAB and *ΔhadABC*/pB strains). Complementation with a plasmid expressing *hadC* restored the wild-type rate of biofilms development (*ΔhadC*/pC,*ΔhadABC*/pBC). At day 7, biolfims could eventually be observed in the *ΔhadC* mutants, but barely in the *ΔhadAC* mutant (*ΔhadABC*/pB), indicating that, the loss of a functional HadA, if it has no visible effect by itself, could again have an additive effect when combined with the inactivation of *hadC*. Fig 6B displays the results of the sliding motility assays. After seven days of incubation, the *wt* strain cells (*wt*/pGBT, *ΔhadABC*/pABC) covered the whole surface of the Petri dish, whereas cells bearing a *ΔhadC* deletion (*ΔhadC/pGBT* and *ΔhadABC*/pB strains) remained concentrated at the center of the Petri dish. Plasmids expressing *hadC* did restore a wild-type phenotype (*ΔhadC*/pC, *ΔhadABC*/pBC). Again the loss of a functional HadA (*ΔhadABC*/pBC) has no visible effect by itself, but aggravates the *ΔhadC* phenotype (*ΔhadABC*/pB). These results therefore underscore the major role of the accessory gene *hadC* and strengthened the notion that HadC contributes to the hydrophobicity potential of the envelope.

### Resistance to SDS and permeability to the lipophilic Nile Red molecule

**Fig 7:**
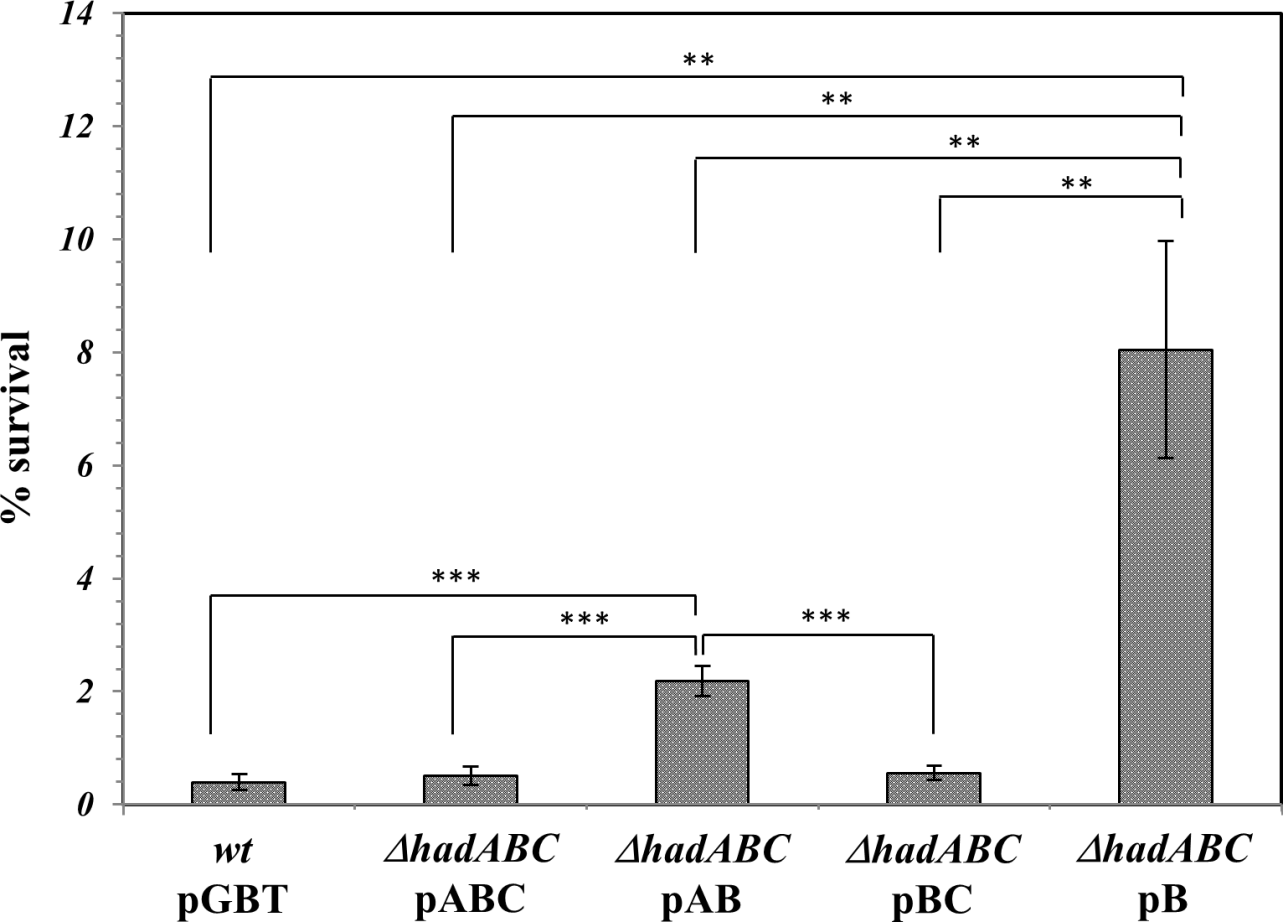
The *hadC* and *hadAC* mutant strains are more resistant to SDS. The survival rates were estimated by counting the CFUs (in duplicate). Error bars represent standard deviation from three biological replicates. P-values < 0.05 (*), < 0.01 (**), < 0.001 (***).

**Fig 8:**
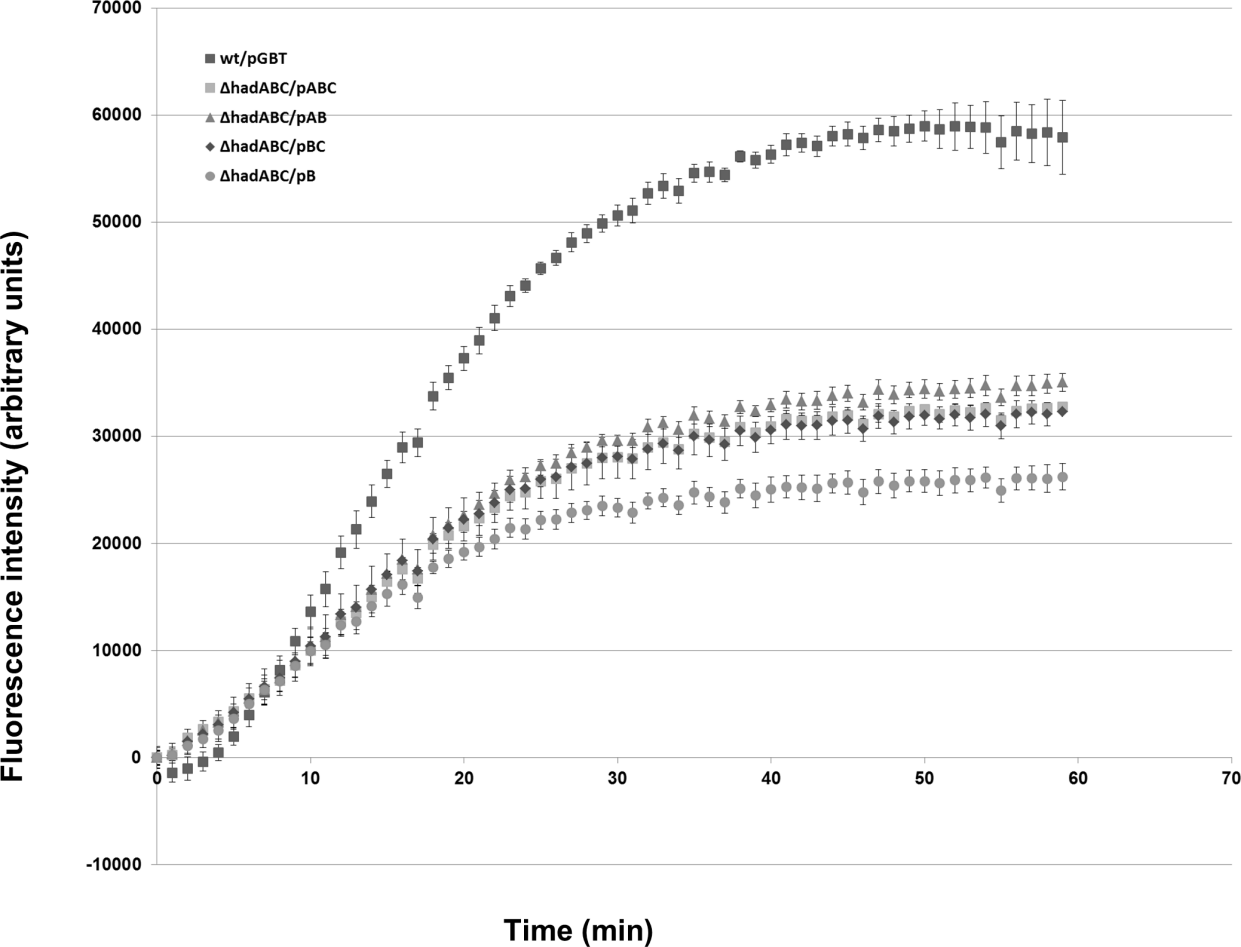
The *hadAC* double mutant envelope is more permissive to the lipophilic Nile Red. This is a representative experiment of two biological repeats giving similar results. Data and error bars from technical triplicate represent the means and standard deviation, respectively.

Alteration of the hydrophobicity of the envelope may be detected by measuring the sensitivity of bacteria to detergents [36,37]. The cell fraction surviving a 65-min challenge to 0.1% SDS was then measured (Fig 7). The survival rate of *wt* strains (*wt*/pGBT, *ΔhadABC*/pABC), as well as that of the *ΔhadA* strain *(ΔhadABC/pBC*), was similar and closed to 0.4%. In contrast the deletion of *hadC (ΔhadABC/pAB)* rendered the bacteria 6-fold more resistant to SDS. When the deletion of *hadA* was combined to that of *hadC (ΔhadABC/pB*), the resistance of the bacteria was even higher, with a survival rate of 8%. These results are in agreement with a lowering of the hydrophobicity of the envelope associated with the inactivation of *hadC* and again confirmed the synergistic effect of the simultaneous loss of *hadA*. With the affected hydrobobicity of the *ΔhadC* envelope, the mutant is expected to accumulate less the lipophilic molecule Nile Red [30]. Accordingly, although the *wt*-like reference strain (*ΔhadABC*/pABC) did accumulate the Nile Red molecule less efficiently that the real *wt* strain (*wt*/pGBT), the accumulation kinetics of the *ΔhadAC* double mutant (*ΔhadABC*/pB) was much less efficient than those of the two former strains (Fig 8). Although with this assay the phenotype of the single mutants (*ΔhadABC*/pBC or *ΔhadABC*/pAB) could not be distinguished from that of the control strain (*ΔhadABC*/pABC), the results reinforced the notion that HadA exacerbates the effect of HadC and that both proteins are required for maintaining the hydrophobic potential of the envelope.

### The fitness of the *ΔhadC* and *ΔhadAC* mutants is compromised at high temperature

**Fig 9:**
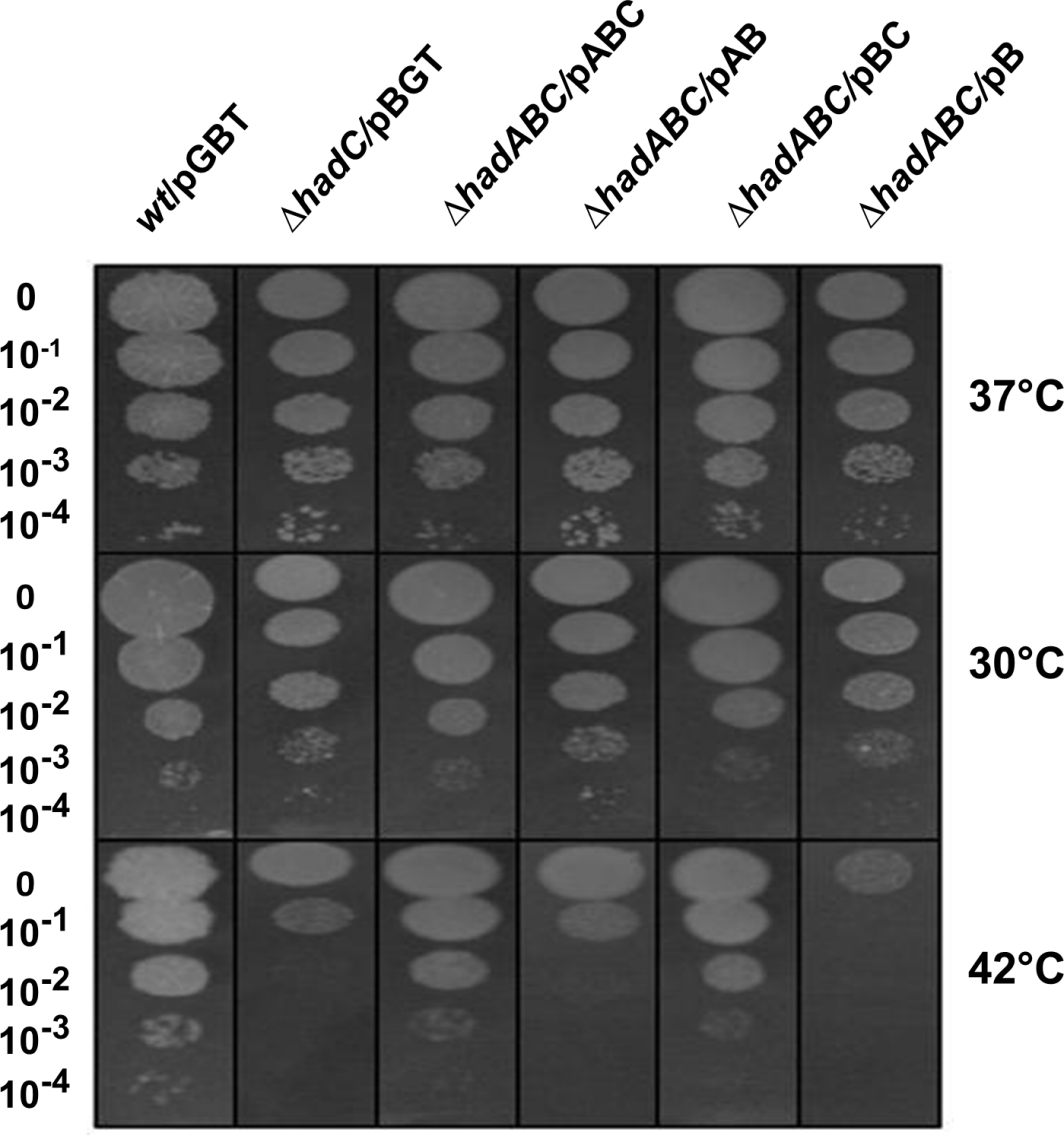
The fitness of the *hadC* and *hadAC* mutants is compromised at high temperature. CFUs were recorded at day 4 for the 37°C and 42°C plates and at day 5 for the 30°C plates.

Considering the importance of the thermal regulation of the fluidity of the biological membrane and the contribution of the MA structure to this fluidity in mycobacteria [38,39], we challenged the fitness of the *had* mutants at different temperatures. As shown in Fig 9, the efficiency of colony formation of the *hadC* mutants (*ΔhadC/pGBT*, *ΔhadABC*/pAB) was similar to that of the appropriate *wt* reference strains (wt/pGBT,*ΔhadABC*/pABC) at 30°C and 37°C, but was three orders of magnitude lower at 42°C. The temperature sensitivity of the mutant was fully complemented by the *hadC-*expressing plasmid (*ΔhadABC/pABC)*. A functional HadC is therefore required for optimal growth at high temperature. In contrast the fitness of the *hadA* mutant (*ΔhadABC*/pBC) was not compromised at 42°C. Moreover, when the *hadA* mutation was combined with the *hadC* deletion (*ΔhadABC*/pB), the sensitivity of the double mutant to high temperature was one order of magnitude even higher than that of the single *hadC* mutation (*ΔhadC/pGBT*, *ΔhadABC*/pAB). Again a phenotype associated to the inactivation of *hadA* was only visible in a *ΔhadC* background.

### Sensitivity of the *ΔhadC* mutant to selective drugs

The very low permeability of the envelope is a hallmark of mycobacteria [40]. This characteristic contributes to the resistance of mycobacteria to a wide range of antibiotics [41]. As MAs, the major constituent of the mycomembrane, play an essential role to this barrier [42], we measured the susceptibility of the *had* mutant to various drugs. The results are shown in Table 2. The efficiency of colony formation of the *hadC* mutant (*ΔhadC/pGBT*) towards Rifampicin (RIF), a large lipophilic anti tubercular drug targeting the transcription apparatus was at least three orders of magnitude below that of the *wt* strain (*wt*/pGBT). This susceptibility phenotype was fully complemented by the *hadC* expressing plasmid pC (*ΔhadC/*pC). The loss of HadA neither impaired the susceptibility of the strain (*ΔhadABC*/pBC) nor exacerbated the susceptibility of a *hadC* mutant (*ΔhadABC*/pB) to RIF. The *ΔhadC* mutant (*ΔhadC/pGBT*) was also two orders of magnitude more susceptible to Isoniazid (INH) and Ethionamid (ETH), a first- and a second-line anti tubercular small hydrophilic drugs, respectively, both targeting the FAS-II InhA enzyme [43]. The pC complementing plasmid fully restored the wild-type susceptibility level (*ΔhadC/*pC). Again the loss of a functional HadA was neutral, in both the single (*ΔhadABC*/pBC) and in the double mutants (*ΔhadABC*/pB). Importantly, when challenged against Ethambutol (EMB), another small hydrophilic drug that targets the arabinogalactan biosynthesis machinery [44], no effect of the various mutations was observed. The susceptibility of the mutants was also tested against Vancomycin (VAN), a drug that cannot diffuse across outer membranes, including the mycomembrane [45], due to its size (1450 Da) and its hydrophilicity. Interestingly, the *hadC* mutant was more susceptible to VAN by two orders of magnitude than was the *wt* and the complemented *hadC*/pC strains. This result therefore suggested a dramatic lowering of the hydrophobicity of the mycomembrane. Again the *hadA* mutation alone (*ΔhadABC*/pBC) had no visible effect on the susceptibility to VAN. However when the mutation was combined with the *hadC* deletion (*ΔhadABC*/pBC) the efficiency of plating decreased further down by two orders of magnitude when compared to that of the *ΔhadC* strain.

**Table 2:** Drug hyper-susceptibility of the *had* mutants. CFUs were recorded at day 4 of incubation at 37°C. Relative values to *wt*’s are shown.

Drug (μg/ml)	*wt/pGBT*	*ΔhadC/pGBT*	*ΔhadC/pC*	*ΔhadABC/pABC*	*ΔhadABC/pBC*	*ΔhadABC/pB*
**Ethambutol (5)**	**1**	**1**	**1**	**1**	**1**	**1**
**Isoniazid (5)**	**1**	**10** ^**−2**^	**1**	**1**	**1**	**10** ^**−2**^
**Ethionamid (10)**	**1**	**10** ^**−2**^	**1**	**1**	**1**	**10** ^**−2**^
**Rifampicin (2)**	**1**	**< 10** ^**−3**^	**1**	**1**	**1**	**< 10** ^**−3**^
**Vancomycin (1)**	**1**	**10** ^**−2**^	**1**	**1**	**1**	**10** ^**−4**^

### Mycolic acids profile of the *hadC* mutants

**Fig 10:**
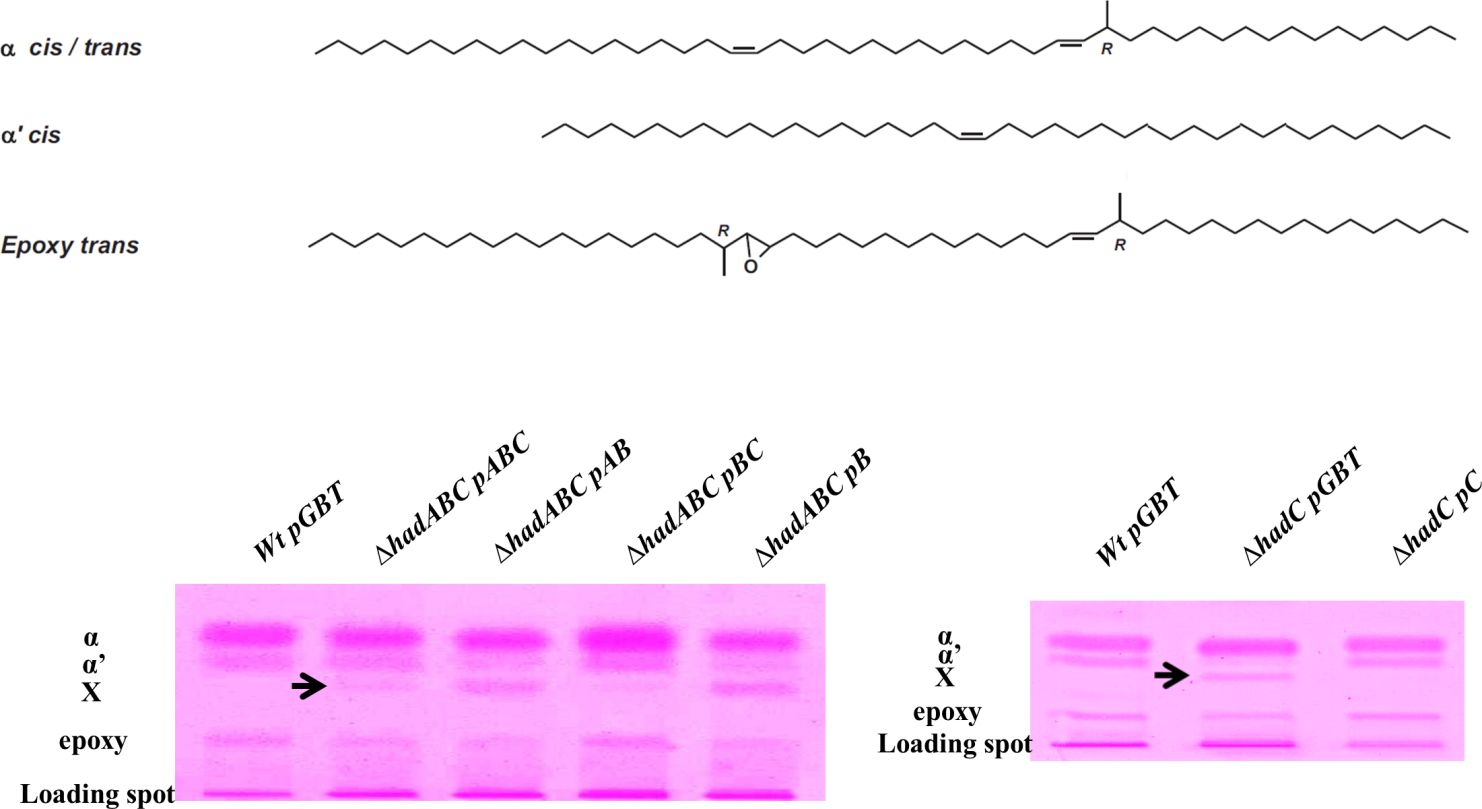
The inactivation of HadC alters the MA profile. (Top) Structures of the major species of the meromycolic chains in *M*. *smegmatis*. α, α’ and epoxy refer to the types of mycolic acids produced by the *wt* strain. *cis* and *trans* correspond to the configurations of the double bonds. R refers to the absolute configuration of the asymmetric carbon bearing the methyl branch. (Bottom) High-performance thin layer chromatography (HPTLC) of total fatty acid methyl esters derived from the saponification of crude cells from the *wt*, isogenic mutant and complemented strains.

Mycolic acids (MAs) are 2-alkyl, 3-hydroxyl fatty acids, composed of a so-called meromycolic chain synthesized by the FAS-II complex, which is condensed with a shorter fatty acid chain that generates the so-called α-branch synthesized by FAS-I. Each mycobacterium species is defined by specific types of meromycolic chains depending on the length of the chain and the types of decorations that are carried by the chains. In order to evaluate the impact of the mutations of the *had* genes on the MA structures, lipids were extracted from the different mutants and analyzed. Similar amounts of lipids were extracted from the strains (roughly 17% of the dry weight). Saponification of the bacterial residues, followed by extraction and methylation of MAs yielded comparable amounts of lipid material (8–9% of the delipidated cell walls), which were quantitatively analyzed by high-performance thin layer chromatography (HPTLC). The *M*. *smegmatis wt* displayed the expected ɑ-, ɑ’-, and epoxy-MAs (Fig 10) [31]. In the *ΔhadABC* background, the complemented *ΔhadABC*/pABC as well as the *ΔhadA* (*ΔhadABC*/pBC) strains displayed a profile similar to that of the *wt* (*wt*/pGBT), with traces of an additional spot (named X on Fig 10). These data are in agreement with the phenotypes described above, *viz* (i) that the pABC plasmid did not fully complement the *ΔhadABC* deletion and (ii) that the single inactivation of HadA had no obvious impact on the bacterial physiology. In contrast, the deletion of *hadC*, both in the *ΔhadC* (*ΔhadABC*/pAB) and *ΔhadAC* (*ΔhadABC*/pB) mutants, clearly altered the HPTLC profile, with a decrease of the amount of epoxy-MAs and a concomitant increase of compound X. A similar altered profile was observed for the simple *ΔhadC* mutant (*ΔhadC*/pGBT) that was complemented by the pC plasmid (*ΔhadC*/pC) (Fig 10).

As shown in Table 3, the relative amount of α-, α’- and epoxy-MAs in the *wt* strain was 63%, 23% and 14%, respectively. In the *ΔhadC* mutant, whereas the sum of α- and α’-MAs (hardly separated by HPTLC) remained similar to that in the *wt* strain (84%), the relative amount of epoxy-MAs decreased to 5% with a concomitant emergence of the compound X representing 11% of the total. Complementing with the plasmid pC (*ΔhadC*/pC) has kept the relative amounts of the α- and α’-MAs close to the wt values and has partially restored percentage of epoxy-MAs (9%) concomitantly to a decrease of compound X (4%).

**Table 3:** Inverse quantitative relationship between the epoxy-MAs and compound X. Lipids were quantitated by high-performance thin layer chromatography (HPTLC). Values are average percentages of technical quadruplicates of total extractable lipids ± σ; nd = not detected.

	α	α’	epoxy	X
***wt***	**63.14 ± 1.89**	**22.50 ± 1.47**	**14.36 ± 0.79**	**nd**
***ΔhadC***	**84.09 ± 0.24** ^a^		**4.94 ± 0.74**	**11.02 ± 0.83**
***ΔhadC/pC***	**68.44 ± 2.08**	**19.27 ± 1.09**	**8.57 ± 0.91**	**3.73 ± 0.52**

^**a**^ Additive percentage of both α- and α’-mycolates; α, α’, epoxy and X as in Fig 10.

**Fig 11:**
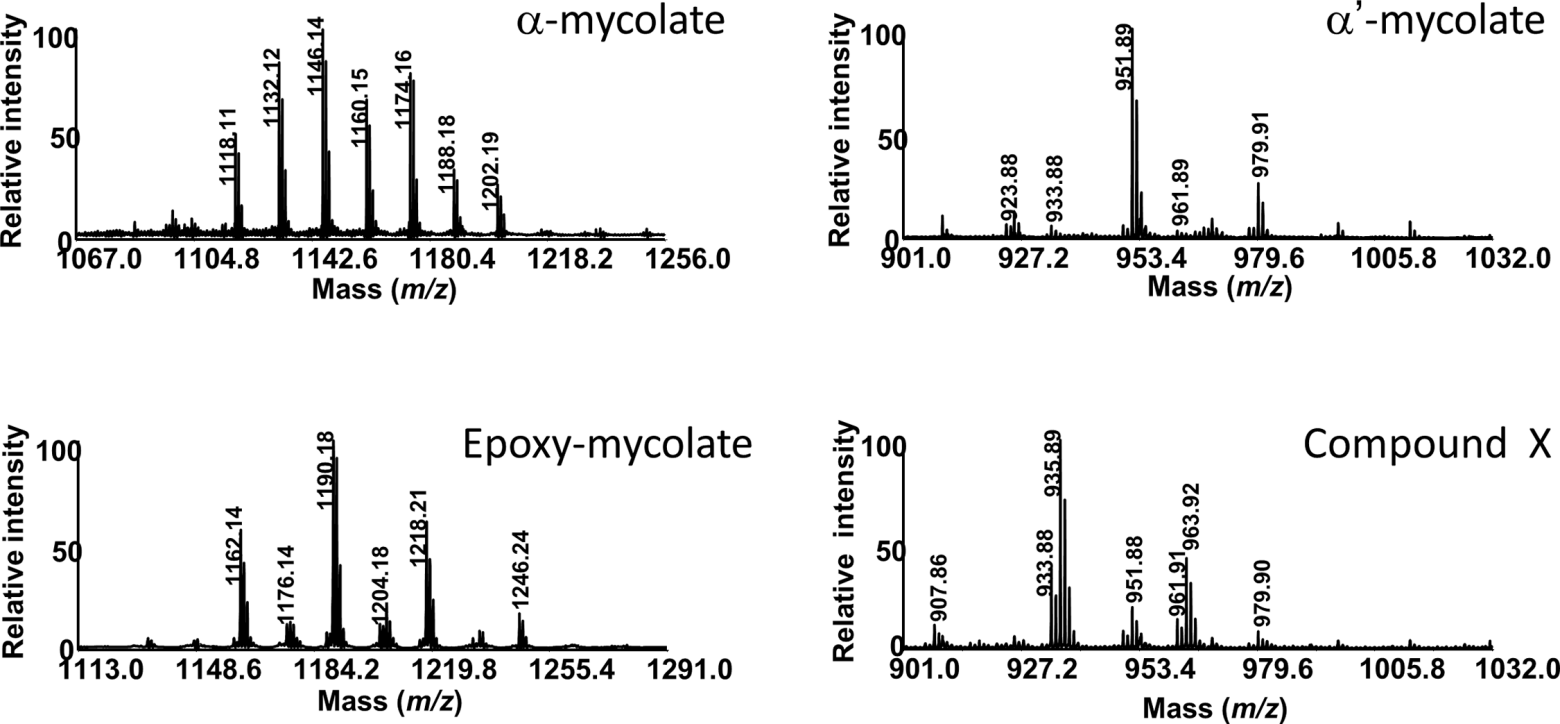
MALDI-TOF mass spectra of the purified compound X, α-, α’- and epoxy-mycolates of *M*.*smegmatis ΔhadC* mutant. Values indicate the masses of sodium adducts (M+23).

MALDI-TOF MS comparative analyses indicated that the mass spectra of α-, α’- and epoxy-MAs from the *wt* and *ΔhadC* mutants displayed similar signals with some differences in the odd/even ratios (data not shown), that may reflect a disruption in the protein-protein interactions between the dehydratases of FAS-II and methyltransferases that introduce unsaturations (double bonds, cyclopropanes) in MAs [28], as recently proposed for *M*. *tuberculosis ΔhadC* mutant [18]. As shown in Fig 11, the mass spectrum of the purified compound X showed [M+Na]^+^ ion-peaks in the mass region of α’-MAs, at *m/z* 907.8 (C59:2), 933. 8 (C61:3), 935.8 (C61:2), 951.8 (C62:1), 961.9 (C63:3), 963.9 (C63:2). NMR analyses indicated similar structures, with the expected resonances [31] for the α-, α’- and epoxy-MAs, whereas the NMR spectrum of compound X did not exhibit the characteristic O-methyl signal from the ester group (at 3.7 ppm), and those at 2.4 and 3.6 ppm, assignable to the proton resonances at C-2 and C-3 positions in MAs, respectively (data not shown). These data therefore indicated the absence of a “mycolic motif” in compound X found chemically highly unstable. Nevertheless, the decrease of the amount of epoxy-MAs observed for the *ΔhadC* mutant (*ΔhadC*/pGBT), which was partially complemented by the pC plasmid (*ΔhadC*/pC), and which was correlated with a concomitant increase of compound X (Fig 10, Table 3) suggests a biosynthetic relationship between the two compounds.

## Discussion

Mycolic acids (MAs), the major components of the mycomembrane, are essential for the viability and/or virulence of pathogenic mycobacteria [13,46]. The essential dehydratase step in the biosynthesis of MAs is performed by the HadA-HadB and HadB-HadC complexes [14,15]. The HadB subunit bears the catalytic domain whereas HadA and HadC determine the substrate specificity [14,47]. In this work we showed, that in *M*. *smegmatis*, the inactivation of *hadC* and *hadA*, both separately and together, was compatible with cell viability. Therefore, either *in vivo* HadB, as a homodimer, could perform the essential dehydration step for the biosynthesis of mycolates or there may be protein(s) that would possess(es) function redundant to those of HadA and/or HadC. The later explanation is supported by phylogenetic studies indicating that Rv0504c (in *M*. *tuberculosis*) and MSMEG_0948 (in *M*. *smegmatis*) are paralogs of HadA and HadC ([28], our unpublished data). Interestingly, a gene encoding a functional HadAB-like complex (MSMEG_6754) has been recently identified in *M*. *smegmatis*, whose overexpression suppresses the lethality of a *hadB* mutation [48]. We have measured the expression of both MSMEG_0948 and MSMEG_6745 in the mutant backgrounds (S1 Fig). Although both genes were actually found down-regulated, one cannot exclude that this level of expression was sufficient to compensate for the loss of HadA and HadC.

Although not essential, the inactivation of *hadC* dramatically impacts the physiology and fitness of the bacterium. The inactivation of *hadA* alone has generally little or no impact in the tested conditions but when combined with the *hadC* deletion, the deletion of *hadA* could exacerbate the phetotype of the *hadC* mutant. The effects were both unspecific and specific. One of the unspecific effects caused by the absence of HadC is the lowering of the hydrophobicity of the cell. Accordingly mutant cells did not aggregate as much as wt cells and did give a smooth colony morphotype, both characteristics usually correlate with an avirulent phenotype in pathogenic mycobacteria species [49,50,51,52]. Similarly, the ability of the *hadC* mutant cells to slide on the surface of growth medium [53] was also impaired, likely because the more hydrophilic envelope of the mutant would interact more with the substratum, preventing the sliding [35]. Similarly, the ability of the *hadC* mutant to develop biofilms was also affected, probably due to the reduced capacity of bacteria cells to aggregate. Although the biological role of biofilms for the pathogenicity in mycobacteria is still an issue [54,55,56], there is a correlation between the structure of MAs, the ability of forming biofilms and the virulence potential [57,58]. Accordingly in *M*. *tuberculosis* H37Rv genetic background we recently observed [18] that the virulence of a *hadC* mutant was compromised as that of the avirulent *M*. *tuberculosis* H37Ra strain that naturally bears a frameshift mutation inactivating the *hadC* [59].

Thermal regulation of the fluidity of biological membranes allows optimal membrane function at different temperatures. This can be done by changing the relative amount and structures of various lipids [39]. Indeed there are several reports showing that MAs structures are regulated by temperature with the ratio of saturation/unsaturation and the relative presence of functional groups in the MAs as well as the chain lengths of MAs being critical determinants of the fluidity of the mycomembrane [38,60,61,62,63]. Our data indicated a change in the sensitivity of the *hadC* and *hadAC* mutants to either cold- or high-temperatures. Expectedly, the MA profile of the *hadC* mutant was clearly altered, with notably the reduction of the synthesis of epoxy-MAs and the concomitant emergence of an unstable molecule (compound X), suggesting a possible biosynthetic filiation between the two compounds. Consistent with our findings, both HadC and HadA subunits have proved to be essential for the solubility and the stability of the HadB-containing enzymes in mycobacteria in vivo [14]. Furthermore, there are evidences for a functional interdependence between methyltransferases involved in the addition of functional groups and that of HadAB/HadBC enzymes [19,28,64,65]. Therefore, in the absence of HadC and HadA subunits, structural modifications that might be crucial to adapt to stresses, such as temperature changes, would no longer be introduced. Alternatively or additionally, as physical interactions between the proteins of the FAS-II system have been reported [65,66], the absence of HadC and HadA subunits might affect the stability of the whole biosynthesis complex in such a way that the synthesis of MAs would be compromised at high temperature.

The alteration of the permeability barrier in the *hadA* and *hadC* mutants was expected to impact the susceptibility of the bacteria to exogenous toxic compounds. Indeed the *hadC* mutant was more susceptible to Isoniazid (INH), Ethionamid (ETH) and Rifampicin (RIF) three anti-tubercular drugs currently used, and to Vancomycin (VAN). The susceptibility of the *hadC* mutant to RIF and VAN was exacerbated by a simultaneous deletion of *hadA*. In contrast, the susceptibility of the mutants to the anti-tubercular drug Ethambutol (EMB) was not changed. VAN is a large hydrophilic molecule that cannot diffuse through the lipophilic outer membrane lipid bilayer and as such cannot be used against gram negative pathogens. The higher susceptibility of the *hadC* and *hadAC* mutants to VAN was likely due to the lower hydrophobicity of their mycomembrane, allowing a better diffusion of the drug to reach its target, the growing peptidoglycan layer [67]. The higher susceptibility to RIF, a large hydrophobic molecule, might be explained by a higher fluidity of the mycomembrane in the mutants, permitting a better global diffusion of the drug [42,68]. Indeed, the inactivation of the *hadC* gene in *M*. *tuberculosis* results in the production of shorter MAs with higher unsaturation degree, two characteristics promoting an increased fluidity of the mycomembrane [18]. Interestingly, although EMB, INH and ETH are all small hydrophilic molecules, the *hadC* mutant was more susceptible to INH and ETH only. As this type of molecules likely diffuses through porins [42,69,70], the efficiency of their penetration into the cell would be less sensitive to the variation of hydrophobicity of the mycomembrane. Therefore the differential response of the mutant regarding the three drugs is probably more related to their modes of action. EMB targets the arabinogalactan biosynthesis pathway [44] whereas INH and ETH target InhA, an enzyme of the FAS-II complex [43]. The absence of HadC might affect the overall structure of the FAS-II complex allowing a better accessibility of InhA to the drugs.

The low fitness as well as the higher susceptibility to drugs of the *hadC* and *hadAC* mutants suggest that targeting HadC and HadA could make the bacterium more susceptible to the natural host defenses and potentiate the activity of current anti-tubercular drugs. The outcome would be the possibility of reducing the drug dosage, limiting therefore the toxic side-effects as well as a shortening of the treatment. *In fine* that should improve the poor compliance with medical treatments, the main reason for the occurrence of resistant strains [8,71]. Actually, Thioacetazone and Isoxyl drugs both target HadAB enzyme and possibly HadBC [64,72,73]. They were both used as anti-tubercular agents but barely now because of a low efficacy (for Isoxyl [20] or toxic side-effects (for Thioacetazone [21]). However, more efficient and less toxic derivatives of both drugs have been reported [72,74,75]. In addition using the *hadC* and/or *hadAC* mutant(s) instead of the *wt* strain in an exhaustive drug screening might lead to the discovery of not only new anti-tubercular drugs but also to drugs already used against other pathogens that might also be now efficient against *M*. *tuberculosis*. Such a strategy based on the targeting of non-essential genes to weaken bacteria might be applied to any pathogens, as well.

## Supporting Information

S1 Fig Relative expression of the *hadA*, *hadB*, *hadC*, *MSMEG_0948 and MSMEG_6754* genes in the mutant strains. Error bars are SEM from three biological triplicates. The expression (log2) in the different mutants was relative to the *wt* strain values (*wt*/pGBT) and measured by RT-qPCR. For each sample, 1 μg of RNA was reverse transcribed using random hexamers and Supercript III reverse transcriptase (Invitrogen) according to the manufacturer’s instructions. qPCR on purified cDNA was performed using KAPA SYBRFAST qPCR Master Mix universal (CliniSciences) and appropriate primer sets (S1 Table). qPCR in technical duplicate was performed in a Bio-Rad CFX96 thermocycler with the following protocol: denaturation at 95°C for 3 min, followed by 40 cycles of denaturation at 95°C for 3 s and annealing/elongation with data collection at 60°C for 20 s. Standard curves and melting curves were drawn to check for the amplification efficiency and the specificity of each primer pairs, respectively. The mean threshold cycle (CT) value was normalized against *sigA* CT. The fold difference of expression was calculated using the Pfaffl method (Pfaffl MW (2001) A new mathematical model for relative quantification in real-time RT-PCR. Nucleic acids research 29: e45).
(TIF)

S1 Table List of the RT-qPCR primers. (DOCX)
