# Supplementary figures and images for: The Non-Essential Mycolic Acid Biosynthesis Genes hadA and hadC Contribute to the Physiology and Fitness of Mycobacterium smegmatis
Source: PLoS One. 2015 Dec 23;10(12):e0145883. doi: 10.1371/journal.pone.0145883 (PMC4689354; doi:10.1371/journal.pone.0145883)

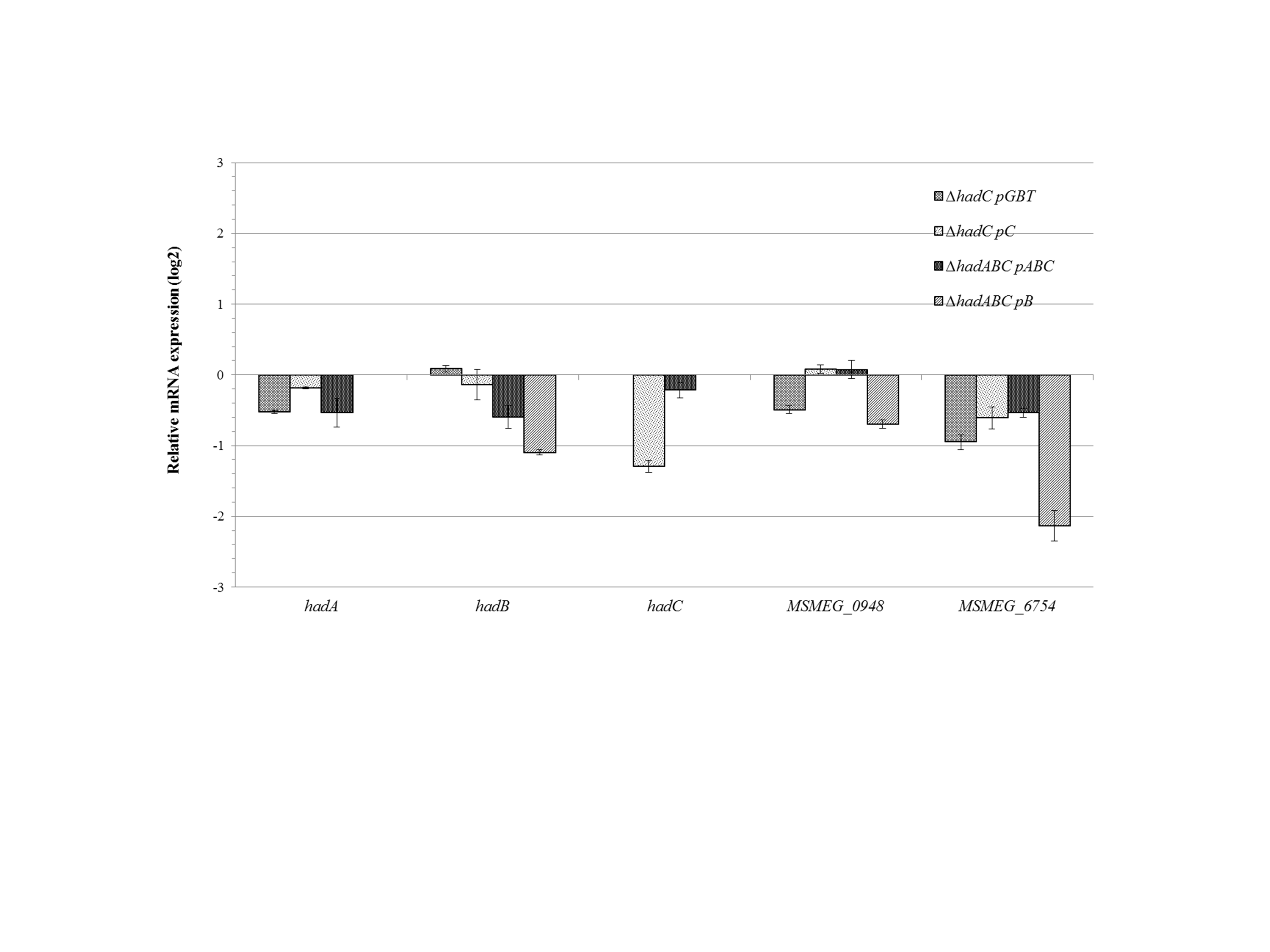

Supplement: S1 Fig — Error bars are SEM from three biological triplicates. The expression (log2) in the different mutants was relative to the wt strain values (wt/pGBT) and measured by RT-qPCR. For each sample, 1 μg of RNA was reverse transcribed using random hexamers and Supercript III reverse transcriptase (Invitrogen) according to the manufacturer’s instructions. qPCR on purified cDNA was performed using KAPA SYBRFAST qPCR Master Mix universal (CliniSciences) and appropriate primer sets (S1 Table). qPCR in technical duplicate was performed in a Bio-Rad CFX96 thermocycler with the following protocol: denaturation at 95°C for 3 min, followed by 40 cycles of denaturation at 95°C for 3 s and annealing/elongation with data collection at 60°C for 20 s. Standard curves and melting curves were drawn to check for the amplification efficiency and the specificity of each primer pairs, respectively. The mean threshold cycle (CT) value was normalized against sigA CT. The fold difference of expression was calculated using the Pfaffl method (Pfaffl MW (2001) A new mathematical model for relative quantification in real-time RT-PCR. Nucleic acids research 29: e45). (TIF) [file pone.0145883.s001.tif]
