# Supplementary material for: The Non-Essential Mycolic Acid Biosynthesis Genes hadA and hadC Contribute to the Physiology and Fitness of Mycobacterium smegmatis
Source: PLoS One. 2015 Dec 23;10(12):e0145883. doi: 10.1371/journal.pone.0145883 (PMC4689354; doi:10.1371/journal.pone.0145883)
Supplement: S1 Table — (DOCX) [file pone.0145883.s002.docx]

**S1 Table. List of RT-qPCR primers**

| Primers | Sequence 5’ 🡺 3’ |
| --- | --- |
| MS_hadA_dir | tctgcatcttcggctatcag |
| MS_hadA_rev | ccttgatcggcttgaagaac |
| MS_hadB_dir | cgtgagttcagttcggtcaa |
| MS_hadB_rev | agtggatcgggttgaggtc |
| MS_hadC_dir | caagggaatggtctggaagt |
| MS_hadC_rev | gcatgaagaagtgctgctg |
| MS_sigA_dir | aagacaccgacctggaactc |
| MS_sigA_rev | cggcatcagcttcttcttc |
| MS_0913_dir | tgatcggcatcacccttag |
| MS_0913_rev | cgttgaactcttcccaacc |
| MS_1350_dir | gtggtcatggagcaggaga |
| MS_1350_rev | cttgtcgtagcggtcgtaga |
| MS_1351_dir | gagcggtacgacgacttctt |
| MS_1351_rev | ggtgacgatgaacctgatga |
| MS_1205_dir | tgaaagaaacgccacagaaa |
| MS_1205_rev | taggcgcaactgtaggtctg |
| MS_1203_dir | tcacgcggttcatctacaag |
| MS_1203_rev | gtcgagcgtgcgaacatag |
